# Structural and Thermoanalytical Characterization of 3D Porous PDMS Foam Materials: The Effect of Impurities Derived from a Sugar Templating Process

**DOI:** 10.3390/polym10060616

**Published:** 2018-06-05

**Authors:** José González-Rivera, Rossella Iglio, Giuseppe Barillaro, Celia Duce, Maria Rosaria Tinè

**Affiliations:** 1 Department of Information Engineering, University of Pisa, via G. Caruso 16, 56122 Pisa, Italy; jose.gonzalezrivera@for.unipi.it (J.G.-R.); rossella.iglio@ing.unipi.it (R.I.); 2 Department of Chemistry and Industrial Chemistry, University of Pisa, Via Moruzzi 3, 56124 Pisa, Italy; mariarosaria.tine@unipi.it

**Keywords:** PDMS, sugar templating process, 3D porous network, thermal stability, TG-FTIR, X-ray (Micro-CT) microtomography

## Abstract

Polydimethylsiloxane (PDMS) polymers are extensively used in a wide range of research and industrial fields, due to their highly versatile chemical, physical, and biological properties. Besides the different two-dimensional PDMS formulations available, three-dimensional PDMS foams have attracted increased attention. However, as-prepared PDMS foams contain residual unreacted low molecular weight species that need to be removed in order to obtain a standard and chemically stable material for use as a scaffold for different decorating agents. We propose a cleaning procedure for PDMS foams obtained using a sugar templating process, based on the use of two different solvents (hexane and ethanol) as cleaning agents. Thermogravimetry coupled with Fourier Transform Infrared Spectroscopy (TG-FTIR) for the analysis of the evolved gasses was used to characterize the thermal stability and decomposition pathway of the PDMS foams, before and after the cleaning procedure. The results were compared with those obtained on non-porous PDMS bulk as a reference. Micro-CT microtomography and scanning electron microscopy (SEM) analyses were employed to study the morphology of the PDMS foam. The thermogravimetric analysis (TGA) revealed a different thermal behaviour and crosslinking pathway between bulk PDMS and porous PDMS foam, which was also influenced by the washing process. This information was not apparent from spectroscopic or morphological studies and it would be very useful for planning the use of such complex and very reactive systems.

## 1. Introduction

Polydimethylsiloxanes are organosilicon polymers commonly used in a wide range of industrial, biomedical, and medicinal or pharmaceutical applications, either in pure form or as formulations. Their structural features (Si–O–Si angles; Si–O bond length, dissociation energy, and freedom of rotation; weak intermolecular forces) make them very flexible polymers with unique physical and chemical characteristics [1,2]. They exhibit low glass transition temperatures (*T*_g_), good resistance to thermal and oxidative degradation, good permeability to gas, and good dielectric properties. Their low surface tension makes them excellent surface active agents. They are also biocompatible, with low toxicity, and therefore suitable for many physiological and biomedical purposes [2].

Different types of PDMSs are used for various applications, such as silicone oils, bulk PDMS, and porous PDMS.

Commercially available silicone oils are typically used as protective coatings for industrial substrates, thanks to their chemical and physical properties as well as their ability to form thin films. They can be easily modified to form hybrid, nano-composite materials suitable for use as anticorrosives [3], ice-retarding [4], self-cleaning/antireflective [5], and, flame/heat/fire retardants [6], or in enzyme immobilization to obtain biocatalytic paints with antifouling/antibiofouling properties [7].

Bulk PDMS is widely used in biomedical applications, thanks to its good blood compatibility, low toxicity, and good thermal and oxidative stability. Medical devices made in PDMS include mammary prostheses [8], cell bioreactors [9], contact lenses [10], and microfluidic devices [11]. In addition, the relatively low Young’s modulus of bulk PDMS (0.4 MPa) has led to the successful use of this soft polymer in the replica molding technique, for the fabrication of patterns with features on a micro and nano-scale [12,13].

More recently, three-dimensional (3D) PDMS foams, namely, porous PDMS, have attracted attention in areas such as medicine, chemistry, materials science, and engineering. Due to its unique properties and easy fabrication, porous PDMS has been used in many applications, such as oil/water separation [14,15,16,17], cellular scaffolds [18], microfluidic pumps [19,20,21], and stress strain sensors [22,23].

3D PDMS foams with either ordered or random porous skeletons have been reported. For instance, Duan et al. used a 3D printing technique to prepare regular, porous polylactic acid scaffolds which were used as a template to create 3D ordered porous PDMS foams. The 3D PDMS was then integrated with a carbon nanotube/graphene network to obtain a stretchable strain sensor [23]. 3D PDMS foams with a random porous skeleton are easily fabricated by replicating the structure of a different kind of random, porous sacrificial template. For instance, Chen et al., used a nickel foam as a 3D template. They replicated its architecture by impregnation method using diluted PDMS (as a solution), achieving a highly porous framework with continuous macropores [24].

Several materials, such as common sugar cubes or grains, solid particles of citric acid monohydrate (CAM), and salts (like NaCl) can be used for the easy, low cost, and eco-friendly preparation of porous PDMS [14,15,17,18,21,22]. These preparation methods should meet two chief criteria: (1) solvent wettability to PDMS and, (2) template solubility in the solvent [17]. The most commonly used solvents are ethanol and water.

A blend of PDMS prepolymer and sacrificial material is usually prepared, which is casted and polymerized on a suitable template, and the sacrificial material is then dissolved. The time required to remove the sacrificial materials can be quite long, especially when its concentration is not high enough to ensure the formation of a connected domain within the polymer. For instance, Yu et al., prepared a porous PDMS sponge for oil/water separation by directly mixing CAM particles with PDMS prepolymer. After polymerization, the samples were immersed in ethanol for 6 h to remove the CAM particles [17]. The authors thus obtained a PDMS sponge with an excellent 3D interconnected porous structure and high oil/water separation efficiency. Li et al., obtained PDMS-based 3-D scaffolds containing interconnected micro- and macro-pores for tissue engineering applications, by blending a dispersion of ethanol and NaCl particles with a PDMS prepolymer. To remove the NaCl particles, the samples were immersed for three days in water [18]. Zhang et al., prepared a PDMS oil absorbent using a sugar template method, directly mixing the sugar particles with PDMS prepolymer and p-Xylene. After polymerization, the sugar particles were dissolved with warm water and the p-Xylene was removed with ethanol [16].

The use of pre-formed sacrificial templates, such as sugar cubes, facilitates the infiltration of pure PDMS prepolymer completely within the template and, in turn, a faster template dissolution after polymerization, for example using warm deionized water [15,16,21,22]. In fact, the pre-formed sacrificial template ensures a connected path for the sacrificial material, which speeds up sugar dissolution.

The aim of this work was to develop a cleaning procedure for 3D porous PDMS foam obtained using a sugar templating process in order to achieve a standard and chemically stable material to be used as a scaffold for different decorating agents. In fact, according to our experimental experience, the treatment of PDMS foam with hot water is often not enough to obtain a chemically stable PDMS. Residues of sugar, unreacted base and curing agents, low molecular weight oligomers may be introduced in the foam which then lead to a continuous mass loss of the foam during the deposition of various decorating agents by drop-casting from hexane or ethanol.

We propose two solvents as cleaning agents: hexane and ethanol. The cleaning level of the foam was assessed by analyzing the composition of the waste washing solvents by Attenuated total reflectance-Fourier transform infrared (ATR-FTIR) spectroscopy. The morphology of the PDMS foam was characterized by SEM analysis and X-ray micro-CT microtomography. The thermal stability and thermal decomposition pathway of the foam were assessed by thermogravimetry.

The study of thermal decomposition behaviour has been mainly carried out in the literature for bulk PDMS with a different level and type of cross-linker agents [25], different molecular weights [26], or catalyst free PDMS [27].

The thermal degradation of PDMS has been mostly investigated under inert atmosphere, which results in depolymerization, through the Si–O bond scission, leading to the formation of a mixture of different cyclic oligomers as degradation products. In air, CO_2_ and water are also present [28]. Camino et al., showed that the products of thermal degradation of PDMS are influenced by the temperature and heating rate [29]. At high temperatures, a radical mechanism occurs, through homolytic Si–CH_3_ scission. The formation of macro-radicals leads to cross-linking with the formation of ceramic silicon-oxicarbide.

On the other hand, the thermal stability of PDMS is also influenced by the presence of impurities (even at a trace level). If traces of oxygen, moisture, or terminal hydroxyl groups are present in the PDMS scaffold, thermal depolymerization under inert atmosphere occurs at different lower temperatures and produces various decomposition products [27].

There is therefore a need to assess the thermal degradation behaviour and products of decomposition of 3D porous PDMS under different conditions.

In our work, we used thermogravimetry coupled with FTIR (TG-FTIR) for the analysis of the evolved gasses in order to characterize the thermal stability and decomposition pathway of porous PDMS foam before and after the washing procedure. The same cleaning procedure was also applied to PDMS bulk samples as a reference, and the results on a porous and non-porous material were compared.

## 2. Materials and Methods

### 2.1. Materials

Common sugar cubes (Dietor vantaggio), PDMS, Sylgard 184 base and thermal curing agent (containing a Pt-based catalyst) were purchased from Dow Corning Corporation (Wiesbaden, Germany). Hexane (>95%) and ethanol (99.8%) were purchased from Sigma-Aldrich (Milan, Italy) and used as received.

### 2.2. Preparation of Porous PDMS Foam

The 3D porous PDMS framework was prepared according to the methodology reported in [22,30]. Briefly: a sugar cube was placed in a Petri dish containing a mixture of PDMS prepolymer using a 10:1 ratio of base: curing components by weight. The Petri dish was then put in a vacuum chamber for 2 h to allow for complete PDMS capillary infiltration into the sugar template and to remove trapped air bubbles. Sugar cubes infiltrated with the PDMS prepolymer mixture were placed in a convection oven for 4 h at 65 °C to ensure full PDMS polymerization through thermal curing. After the in situ polymerization, the PDMS-sugar templating scaffold composite was then placed in a freezer for 3 min to enable PDMS detachment from the scaffold. Sugar scaffold templates were dissolved by rinsing in deionized water at 60° for 1 h. The resulting 3D porous PDMS foam was dried under a chemical hood for 1 h at room temperature.

The purification steps on both bulk PDMS and 3D porous PDMS were performed as follows: the samples were soaked and stirred in the solvent (ethanol or hexane) for 72 h and the free solvent removed by filtration. The swollen polymers were weighed and the swelling capacity determined according to: % wt. = 100 × [(weight of PDMS_swollen_ − weight of PDMS_dried_)/weight of PDMS_swollen_]. The polymers were then dried at 70 °C for at least 4 h. A second cleaning step was then performed, soaking and stirring the polymeric materials with fresh solvent for 12 h. The swelling capacity was recorded at each step of the soaking/swelling/drying purification process and a mean value was reported. Both the waste solvents and the dried PDMS-based polymers, were analysed by ATR-FTIR spectroscopy (Agilent Technologies, Milan, Italy).

### 2.3. Morphological Characterization

The cross-sections of PDMS foam were investigated using a scanning electron microscope (SEM, JEOL JSM-6390, Milan, Italy) at an accelerating voltage of 5 kV. μ-CT three-dimensional reconstruction was performed using a SkyScan 1174 system (Skyscan, Aartselaar, Kontich, Belgium) with a resolution of 6.5 μm·pixel^−1^, 180° rotation.

### 2.4. Thermogravimetry

A TA Instruments Thermobalance model Q5000IR equipped with an FTIR (Agilent Technologies, Milan, Italy) spectrophotometer Cary 640 model for evolved gas analysis (EGA) was used. TG-FTIR measurements were performed at a rate of 20 °C/min, from 30 to 900 °C under nitrogen flow (70 mL/min) using Pt crucibles, from 600 to 4000 cm^−1^ with a 4 cm^−1^ width slit. A background spectrum was taken before each analysis in order to zero the signal in the gas cell and to eliminate the contribution due to the amount of ambient water and carbon dioxide. Mass calibration was performed using certified mass standards, supplied by TA Instruments, in the range from 0 to 100 mg. The amount of sample in each experiment varied between 10 and 12 mg. Temperature calibration was based on the Curie point of paramagnetic metals. A multipoint calibration with five Curie points from reference materials (Alumel, Ni, Ni83%Co17%, Ni63%Co37%, Ni37%Co63%) was performed.

### 2.5. ATR-FTIR Analysis

Infrared spectra were recorded using an FT-IR Agilent Technologies Spectrophotometer model Cary 640 (Agilent Technologies, Milan, Italy), equipped with a universal attenuated total reflectance accessory (ATRU). A few micrograms of sample powder were used, and for the liquid samples, 20 μL were dripped onto the ATR accessory with the following spectrometer parameters; resolution: 4 cm^−1^, spectral range: 600–4000 cm^−1^, number of scans: 128. Agilent spectrum software (Agilent Technologies, Milan, Italy) was used to process the FTIR spectra. The FTIR spectra of the impurities obtained in the washing solvents were recorded after solvent evaporation.

## 3. Results and Discussion

### 3.1. PDMS Crosslinking Polycondensation Reaction

**Scheme 1:**
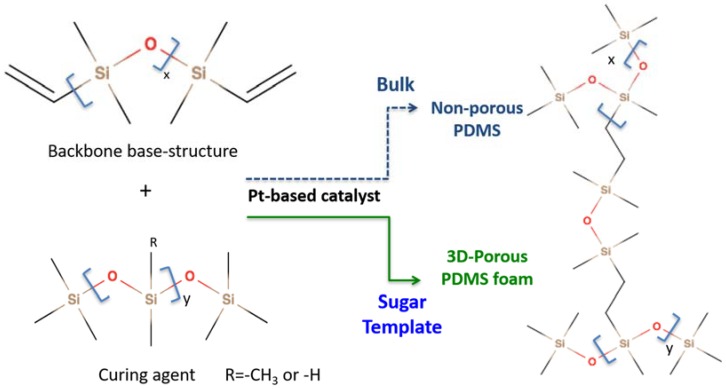
The PDMS crosslinking polymerization reaction.

The Pt-catalysed crosslinking polycondensation reaction occurring between silane terminations (Si−H), present in the PDMS curing and, vinyl groups in the PDMS base precursor (Scheme 1) was used to prepare two different PDMS-based materials: (i) a highly porous PDMS foam with a 3D interconnected macropore framework promoted by the sugar templating process and, (ii) a non-porous crosslinked bulk PDMS.

The FTIR spectra of the curing agent, PDMS base precursor, and the two PDMS polymers (i.e., porous and non porous) materials prepared in this work are shown in Figure S1. The fingerprint of silicone (–Si–O–Si– and –Si–CH_3_ signals, see Table 1 and Figure S1) are evident in the FTIR spectra of bulk PDMS and 3D porous PDMS foam. The main signals corresponding to Si−H (2158 cm^−^^1^ stretching, see Table 1 and Figure S1) present in the PDMS curing agent were not detected for either of the PDMS polymer materials, thus confirming that the crosslinking polymerization reaction was successfully carried out.

However, a small signal at 910 cm^−1^ was detected (see Figure S1) for both bulk PDMS and 3D porous PDMS foam. This peak can be ascribed to a small amount of residual non-cross-linked curing agent entrapped in the framework. In fact, the as-prepared materials can contain residual unreacted low molecular weight species [31] that need to be removed to obtain a standard and stable, either porous or non-porous PDMS, which can be used as a scaffold of different decorating agents.

### 3.2. Surface and Morphological Characterization of 3D Porous PDMS Foam

**Figure 1:**
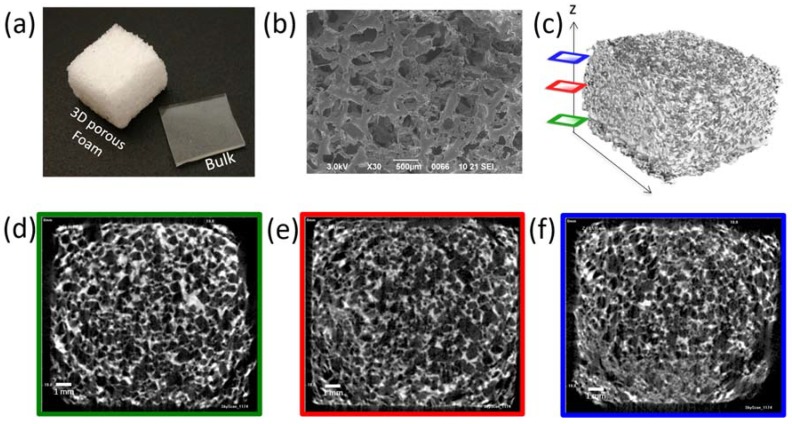
Morphological characterization of the 3D porous PDMS foam obtained by the sugar templating process: (**a**) Optical picture, (**b**) SEM image and, (**c**) 3D volume reconstruction and (**d**–**f**) cross-sectional profiles at different heights (z_b_ = 0.86 mm, z_c_ = 4.12 mm and z_d_ = 8.60 mm, z_max_ = 9.0 mm) obtained by micro CT analysis.

The porous PDMS foam obtained by the sugar templating approach has very complex interconnected pore channels with a widely polydisperse volume along the different dimensions and an optical picture of the as prepared materials is shown in Figure 1a. The PDMS was infiltrated around the sugar grains and after the sugar templating removal, the SEM analysis highlighted that the 3D porous structure had a smooth internal surface area with a macro-pore size in the range of 500 ± 300 µm (Figure 1b).

The porosity of this material has recently been reported (about 77%) and the pore sizes were in agreement with the size of the SEM analysis and those of the sugar grains used as the template [22]. Useful morphological information about different polymeric materials has reported by SEM imaging [32,33].The SEM analysis is however limited to two-dimensional views of a material, which provide insights into the surface textural properties and morphology.

The analysis of the pore size and pore size distribution of materials with a 3D interconnected channel framework, such as the 3D porous PDMS foam fabricated here, is complex and it is thus more appropriate to refer to a pore volume density. The 3D reconstruction of porous PDMS foam performed by the micro CT x ray scanning is shown in Figure 1c. The analysis of cross-sectional slides (see Figure 1d–f), here shown as an example of the interconnected pores at different heights: z_b_ = 0.86 mm, z_c_ = 4.12 mm and z_d_ = 8.60 mm) and the video (showing the whole volume and pore volume evolution along the 3D directions of the porous PDMS foam) obtained by the reconstruction of 475 cross-sectional slides along the Z dimension (see VS1) highlighted that the pore framework in the 3D porous PDMS foam, is an interconnected three-dimensional channel system with a pore volume density gradient that is widely distributed along the various directions. Micro CT X ray scanning is thus a very useful tool for morphological studies of these complex matrixes, as it gives clear visual information on their entire internal structure.

### 3.3. The ATR-FTIR Spectra of the Waste Washing Solvents

Hexane and ethanol were selected as cleaning agents to remove unreacted species. The cleaning level of the materials was assessed by monitoring the presence of waste compounds in the washing solvents by FTIR.

**Figure 2:**
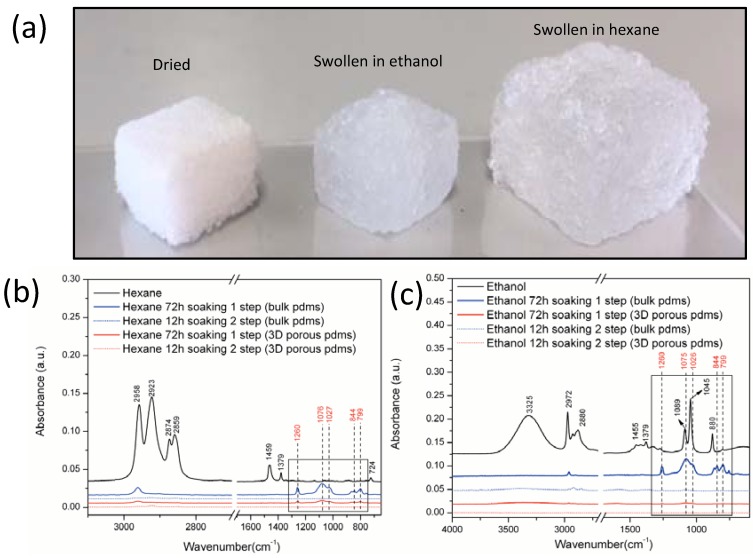
(**a**) A 3D-porous PDMS foam obtained through a sugar-based template approach and its swelling behaviour in ethanol and hexane. ATR-FTIR spectra of hexane (**b**) and ethanol (**c**) cleaning solvents, acquired during the cleaning procedure setup for both bulk PDMS and 3D-porous PDMS foam.

Figure 2a shows the picture of a 3D-porous PDMS foam as-prepared and after it had been swollen in ethanol and hexane. The swelling ratio (or solvent absorption capacity) is as high as 600 wt % in hexane and 300 wt % in ethanol (compared to the weight of the dried 3D-porous PDMS foam). This is significantly higher than that of the bulk PDMS prepared in this work, which was up to 90 wt % in hexane and 11 wt % in ethanol. The improved swelling capacity of 3D-porous PDMS foams with respect to bulk PDMS, can be attributed to the porosity of the material and is in agreement with data for similar PDMS-based materials [17].

The ATR-FTIR spectra of the washing solvents showed that small oligomers from the PDMS base precursor (–Si–CH_3_, –Si–O–Si–, see Table 1 for FTIR assignments) and curing agent (–Si–CH_3_, –Si–H, –Si–O–Si–, see Table 1 for FTIR assignments) were completely removed from both porous and bulk PDMS after 72 h of soaking either in hexane or in ethanol (first purification step, see Figure 2b,c). Mass losses of 4.3% ± 0.1% (hexane) and 3.7% ± 0.3% (ethanol) for bulk PDMS and of 3.8% ± 0.3% (hexane) and 3.4% ± 0.8% (ethanol) for 3D-porous PDMS foam were recorded after the first purification step, with no significant differences for the two solvents.

No significant impurity signals were observed in the FTIR spectra of either the porous or bulk PDMS after a further 12 h soaking in hexane and ethanol (see Figure 2b,c). In this case, the PDMS mass losses were less than 0.1% for both the solvents.

Interestingly, ATR-FTIR spectra of both bulk PDMS and 3D porous PDMS foam acquired before and after soaking and washing in both solvents were unchanged, suggesting that the cleaning procedure does not impact on the PDMS chemical structure (see Figure S2). The FTIR spectra were characterized by –Si–O–Si and –Si–CH_3_ typical absorptions, although Si−H absorption was also visible, suggesting the presence of silane terminal groups in the polymer.

### 3.4. Thermal Behaviour by TG-FTIR Analysis

Thermogravimetric analyses under nitrogen revealed a different thermal behaviour of bulk PDMS and 3D porous PDMS foam, also highlighting various changes induced by the washing process, which were not apparent from the FTIR analysis.

**Figure 3:**
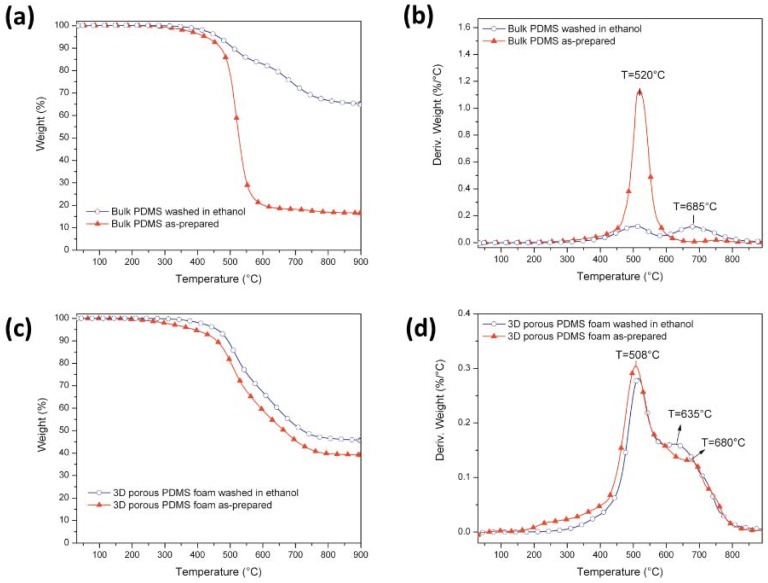
TG and DTG curves under N_2_ of bulk PDMS (**a**,**b**) and 3D porous PDMS foam (**c**,**d**) before and after washing in ethanol.

Figure 3 shows the TG and DTG curves of bulk PDMS (Figure 3a,b) and 3D porous PDMS foam (Figure 3c,d) under N_2_, before and after washing in ethanol.

The as-prepared bulk PDMS showed the typical thermal degradation curve of PDMS [28], with a single sharp mass loss of almost 80% in the temperature range 400–600 °C and a maximum DTG at 520 °C (Figure 3a,b). The as-prepared 3D porous PDMS foam showed a broader mass loss in the temperature range 300–750 °C, with two overlapping steps with maxima at almost 510 and 680 °C.

Washing (both in ethanol and in hexane) produced an increase in the thermal stability of both bulk PDMS and 3D porous PDMS foam, but affected the thermal degradation behaviour of bulk PDMS more than that of 3D porous PDMS foam. The starting temperature of the thermal degradation of both bulk PDMS and 3D porous PDMS foam increased from 300 to 400 °C after washing, confirming the removal of low molecular weight oligomers and unreacted reagents from the materials. Above 400 °C, the thermal degradation curves of 3D porous PDMS foam before and after washing were very similar, in contrast to those of bulk PDMS that are significantly different, with the curve measured after washing becoming more similar to that of 3D porous PDMS foam.

The mass loss spread over the temperature range 400–750 °C, revealing two overlapping steps with a maximum at almost 510 and 680 °C, respectively. The mass loss percentage and residual mass of bulk PDMS after washing presented a high variability (around 15%) ascribed to the intrinsic bulk polymerization approach (yielding a more nonhomogeneous material). The PDMS thermal degradation mechanism proceeds via a depolymerization pathway occurring with two different mechanisms: “unzip degradation” and “rearrangement degradation” [28,29,36]. Unzip degradation generates cyclic siloxanes of different dimensions and occurs at about 400–500 °C. Rearrangement degradation occurs at above 500 °C by heterolytic cleavage and the rearrangement of the Si–O–Si bond in the main chain and generates low molecular weight species and cyclic siloxanes. At 800 °C under inert atmosphere, a black residue ascribed to silicon carbide or silicon oxycarbide is formed [28]. Methane (CH_4_) is the second main byproduct generated by unzip thermal degradation through the homolytic Si–CH_3_ bond scission followed by hydrogen abstraction [29,37]. The production of cyclic siloxanes and the corresponding residues are strongly affected by the cross-linking degree of the starting material and by the further cross-linking reaction occurring in the material during the thermogravimetric measurements [28,29,36].

**Figure 4:**
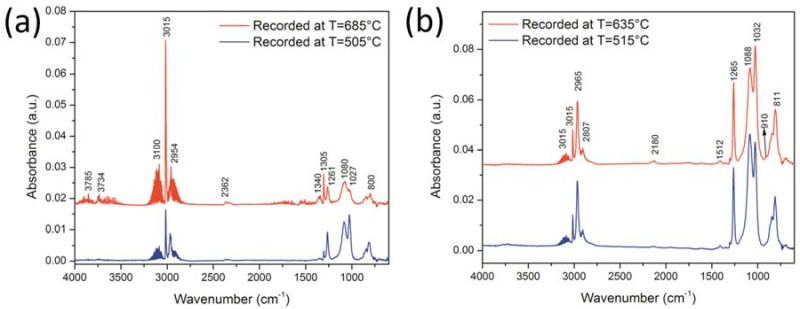
FTIR spectra of evolved gas from (**a**) bulk PDMS and (**b**) 3D porous PDMS foam recorded under N_2_ flow at the two main thermal decomposition steps. Bulk PDMS and 3D porous PDMS foam samples correspond to the ethanol cleaning samples.

To further investigate the thermal decomposition mechanism of both the bulk and porous PDMS materials, the gaseous species evolved during their thermal degradation were analysed by evolved gas analysis (EGA) with FTIR spectroscopy (TG/ FTIR). As an example, Figure 4 shows the FTIR spectra of the gases evolved recorded at 505 and 685 °C for bulk PDMS and at 515 and 635 °C for the 3D porous PDMS foam after cleaning with ethanol. The FTIR spectra of the main degradation step recorded for the as-prepared bulk PDMS and the as-prepared 3D porous PDMS foam are reported in Figure S3. The main compounds identified in the evolved gas during the thermal decomposition of all the tested PDMS-based materials were linear and cyclic siloxane oligomers. The cyclic siloxane oligomers showed two main chemical groups: (1) –Si–CH_3_ (FTIR wavenumber assignments cm^−1^: 813 (Si–C stretching), 1265 (1260 symmetric C–H bending [34]), 1412 (1414 asymmetric C–H bending, [35]), 2967 (2965 asymmetric C–H stretching, [35]), 2904 (2905 symmetric C–H stretching, [35])) and, (2) –Si–O–Si– (FTIR wavenumber assignments cm^−1^: 1027, 1085 due to the asymmetric –Si–O–Si stretching, [35]). Methane was the second chemical species detected (–C–H with FTIR wavenumber assignments of 1303 and 3015 cm^−^^1^, [29,36]).

**Figure 5:**
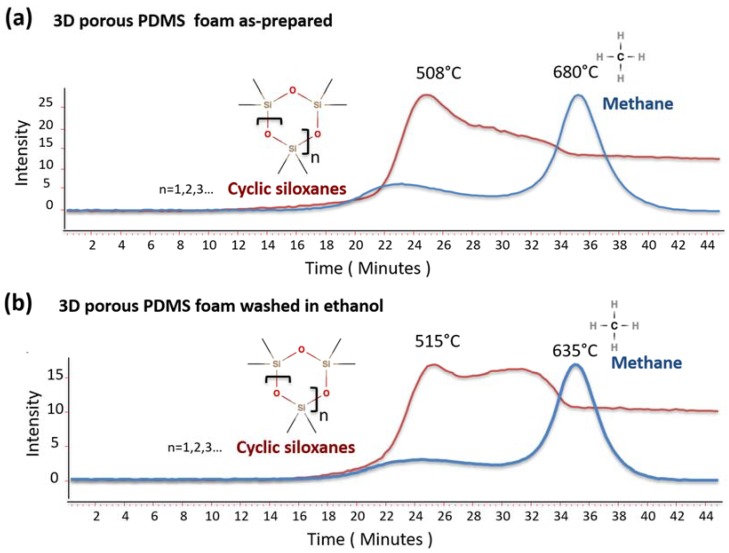
Evolution profiles of CH_4_ and cyclic siloxane compounds from the TG/FTIR of 3D porous PDMS foam thermal decomposition at 20 °C min under N_2_.

**Figure 6:**
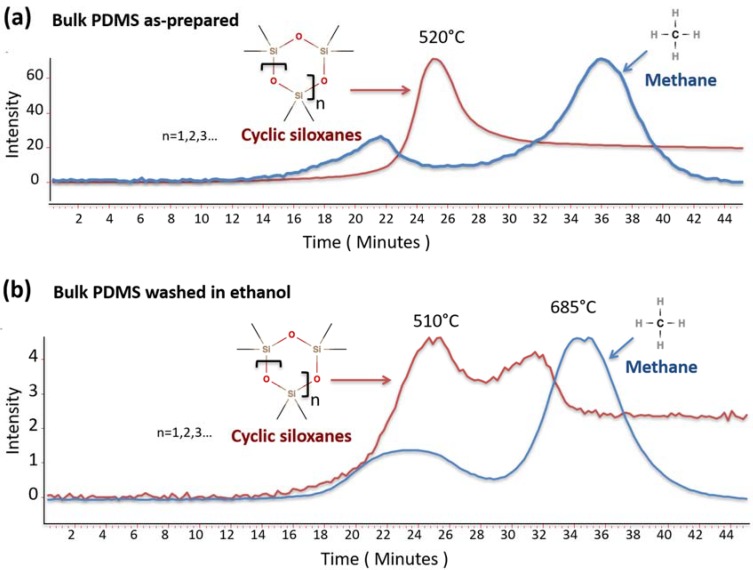
Evolution profiles of CH_4_ and cyclic siloxane compounds from the TG/FTIR of bulk PDMS thermal decomposition at 20 °C min under N_2_.

Figure 5 shows the evolution profiles with the temperature of the main gaseous products (cyclic siloxanes and methane) obtained by monitoring their strongest IR bands for porous PDMS foam before (a) and after (b) washing with ethanol. The curves revealed in both cases two evolution bands of cyclic siloxane compounds, one below 500 °C and the other above 500 °C accounting for both the unzip and rearrangement polymer degradation (see Figure 5). The evolution profiles of cyclic siloxanes for bulk PDMS highlighted a single evolution band, at a temperature below 500 °C, of these compounds for unwashed PDMS and two evolutions after washing. This suggests that the “rearrangement degradation” mechanism started to take place mainly after washing bulk PDMS, see Figure 6.

The CH_4_ evolution was present throughout the degradation time of the tested PDMS materials, though it was predominant at the end of the depolymerization, as the last event of the silicon carbide formation.

In summary, thermogravimetric data and FTIR analyses of the evolved gases suggested that porous PDMS foam is more homogeneous in terms of cross linking and more chemically stable than PDMS bulk.

## 4. Conclusions

Given the increasing interest in porous PDMS foam in the field of sensors, we investigated the chemical composition and thermal degradation of a macroporous PDMS foam obtained using a sugar templating fabrication approach and to find a cleaning procedure based on PDMS washing with solvents to achieve a homogeneous porous PDMS material both in terms of chemical and physical properties. The ATR-FTIR spectra of the washing solvents showed that small oligomers of a PDMS base precursor and curing agent were completely removed from both porous and bulk PDMS. The latter was used as reference, after 72 h of soaking both in hexane and ethanol. Mass losses of 4.3% ± 0.1% (hexane) and 3.7% ± 0.3% (ethanol) for PDMS bulk and of 3.8% ± 0.3% (hexane) and 3.4% ± 0.8% (ethanol) for PDMS foam were registered after the first purification step, and no significant differences between the two solvents (i.e., hexane or ethanol) were observed.

The structural analysis by ATR-FTIR spectroscopy of both bulk PDMS and porous PDMS foam at the different steps in the cleaning procedure highlighted that no significant structural changes, with respect to the as-prepared materials, were induced by soaking and washing the PDMS in the tested solvents.

Interestingly, the thermogravimetric analyses under nitrogen gas revealed a different thermal behaviour between bulk PDMS and porous PDMS foam, which was also influenced by the washing process, especially for bulk PDMS. An increase in the thermal stability of both bulk PDMS and porous PDMS foam was apparent after the washing process, which also caused a modification of the thermal degradation behaviour of both the PDMS materials. In fact, the starting temperature of the thermal degradation of both bulk PDMS and 3D porous PDMS foam increased from 300 to 400 °C, confirming the removal by washing of low molecular weight oligomers and unreacted reagents from the materials. Above 400 °C, the thermal degradation curve of bulk PDMS changed significantly, becoming more similar to that of porous PDMS foam. The evolution profiles of the main gaseous products (cyclic siloxane and methane) obtained by monitoring their strongest IR bands over time for porous PDMS foam, revealed two evolutions of cyclic siloxane compounds. These consisted of one below 500 °C and the other that accounted for both the unzip and rearrangement polymer degradation.

In summary, thermogravimetric data highlighted a modification in the reactive pathway of PDMS materials due to a cleaning procedure. This information, which was not apparent from spectroscopic or morphological studies, would be very useful for planning the use of such complex and very reactive systems.

## Figures and Tables

**Table 1:** Assignments of the main FTIR absorption bands of silicon-based materials.

Wavenumber (cm^−1^)	Assignment	Reference
	Si–CH_3_, Si–CH_2_–	
690, 790	Si–C stretching	[34]
843	C–H (–CH_2_) rocking	[25]
1260	symmetric C–H bending	[34]
1414	asymmetric C–H bending	[35]
2965	asymmetric C–H stretching	[35]
2905	symmetric C–H stretching	[35]
	Si–H	
2158 912	Si–H stretching Si–H bending	[35]
	Si–O–Si	
1023	asymmetric in–Si–O–Si stretching	[25]

## Supplementary Materials

The following are available online at http://www.mdpi.com/2073-4360/10/6/616/s1, Figure S1: ATR-FTIR spectra of bulk PDMS, 3D-porous PDMS foam, PDMS-curing agent and PDMS-base backbone precursors, Figure S2: ATR-FTIR spectra of bulk PDMS (a) and, 3D porous PDMS foam (b) after the materials were submitted to several steps of soaking and washing in hexane and ethanol. ATR-FTIR spectra were recorded after the materials were dried at 70 °C per 4 h, Figure S3: FTIR spectra of evolved gas from (a) bulk PDMS and (b) 3D porous PDMS foam recorded at *T* = 520°C and *T* = 508 °C under N_2_ flow at the main thermal decomposition step, respectively. Bulk PDMS and 3D porous PDMS foam samples correspond to as made samples, Video S1: 3D porous PDMS foam microCT X ray reconstruction.
