# Supplementary material for: Structural and Thermoanalytical Characterization of 3D Porous PDMS Foam Materials: The Effect of Impurities Derived from a Sugar Templating Process
Source: Polymers (Basel). 2018 Jun 5;10(6):616. doi: 10.3390/polym10060616 (PMC6404115; doi:10.3390/polym10060616)
Supplement: Supplementary file 1 [file polymers-10-00616-s001.zip › polymers-310554-supplementary-proofdone/polymers-310554.supplementary.proofdone.docx]

Structural and thermoanalytical characterization of 3D porous PDMS foam materials. The effect of impurities derived from a sugar templating process

José González-Rivera^1^, Rossella Iglio^1^, Giuseppe Barillaro^1, *^, Celia Duce^2,*^ and Maria Rosaria Tinè^2^

^1^ Department of Information Engineering , University of Pisa, via G. Caruso 16, 56122, Pisa,

Italy

^2^ Department of Chemistry and Industrial Chemistry, University of Pisa, Via Moruzzi 3,56124, Pisa, Italy

***** Correspondence: *[g.barillaro@iet.unipi.it](mailto:g.barillaro@iet.unipi.it) (Tel.: +39 050 2217 601); *[celia.duce@unipi.it](mailto:celia.duce@unipi.it) (Tel.: +39 050 2219311).

**
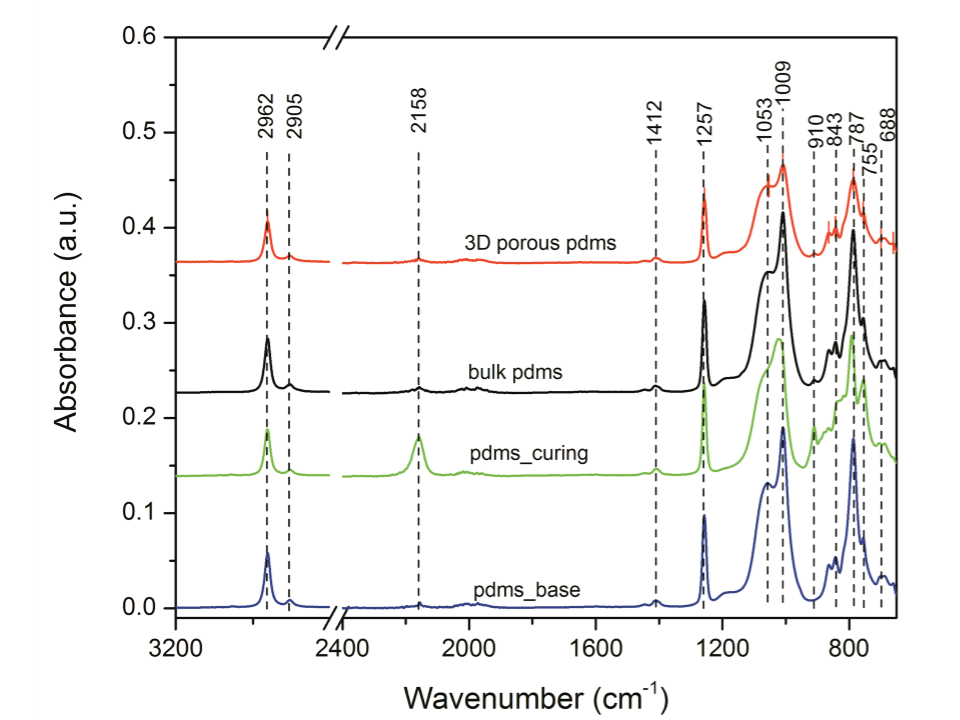
**

**Figure S1**. ATR-FTIR spectra of bulk PDMS, 3D-porous PDMS foam, PDMS-curing agent and PDMS-base backbone precursors.


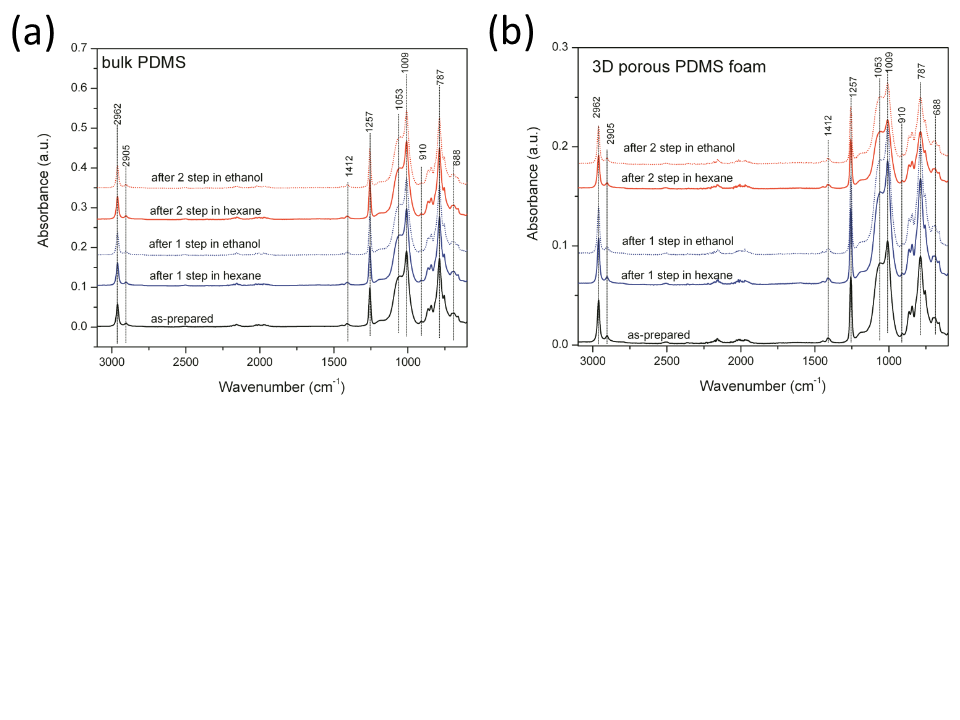


**Figure S2.** ATR-FTIR spectra of bulk PDMS (a) and, 3D porous PDMS foam (b) after the materials were submitted to several steps of soaking and washing in hexane and ethanol. ATR-FTIR spectra were recorded after the materials were dried at 70 °C per 4 h.


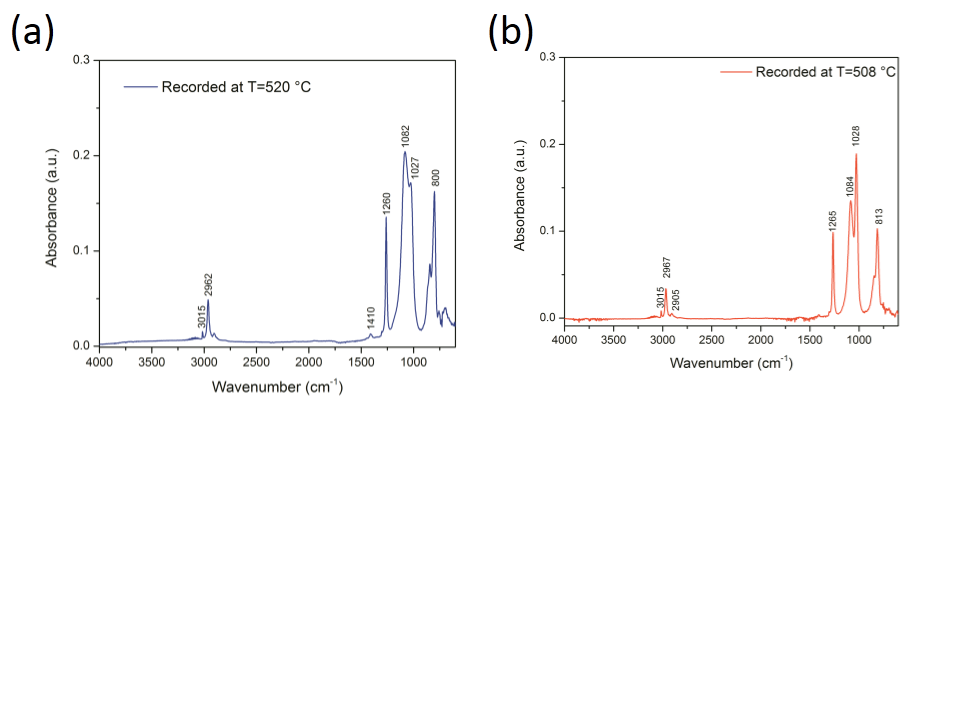


**Figure S3.** FTIR spectra of evolved gas from (a) bulk PDMS and (b) 3D porous PDMS foam recorded at T=520°C and T=508 °C under N_2_ ﬂow at the main thermal decomposition step, respectively. Bulk PDMS and 3D porous PDMS foam samples correspond to as made samples.
